# Lead Exposure Induces Weight Gain in Adult Rats, Accompanied by DNA Hypermethylation

**DOI:** 10.1371/journal.pone.0169958

**Published:** 2017-01-20

**Authors:** Honglin Sun, Ningjian Wang, Xiaomin Nie, Li Zhao, Qin Li, Zhen Cang, Chi Chen, Meng Lu, Jing Cheng, Hualing Zhai, Fangzhen Xia, Lin Ye, Yingli Lu

**Affiliations:** Institute and Department of Endocrinology and Metabolism, Shanghai Ninth People's Hospital, Shanghai JiaoTong University School of Medicine, Shanghai, China; University of Arkansas for Medical Sciences College of Pharmacy, UNITED STATES

## Abstract

**Objective:**

Previous studies have revealed the association of lead (Pb) exposure with obesity. DNA methylation alteration has been suggested to be one of the regulatory mechanisms of obesity. We aimed to explore whether Pb exposure is related with weight gain and DNA methylation alteration.

**Methods:**

Male adult 8 week Wistar rats were divided into 5 groups: the normal chow diet (NCD); the NCD+0.05%Pb; the NCD+0.15%Pb; the NCD+0.45%Pb and the high fat diet. Rats were exposed to different dosages of Pb through drinking water for 21 weeks. Body weight, fasted blood glucose level, fasted insulin level, homeostasis assessment of insulin resistance (HOMA-IR) index and lipid profile were detected. Intra-peritoneal glucose tolerance test (IPGTT) was constructed to evaluate the glucose tolerance. Lipid accumulation of liver was detected and liver DNA underwent whole genome bisulfite sequencing.

**Results:**

The NCD+0.05%Pb group had significantly greater weight, HOMA-IR and triglycerides, and lower glucose intolerance than the NCD group (P <0.05). This group also showed hepatic lipid accumulation. These metabolic changes were not observed in the other two Pb dosage groups. Furthermore, DNA hypermethylation extended along pathways related to glucose and lipid metabolism in NCD+0.05%Pb group.

**Conclusion:**

Pb exposure resulted in dose-specific weight gain in adult Wistar rats, accompanied by alteration of DNA methylation.

## Introduction

The prevalence of overweight and obesity has drawn worldwide attention in all geographic areas during last two decades. In USA, the combined prevalence of overweight and obesity had risen from 14.5% to 35.1% in adults [1], which is similar to growth seen in China from 14.6% to 32.3%, due to economic boom and lifestyle changes [2]. However, environmental factors are attributed to the rising prevalence of obesity, too. Endocrine disrupting chemicals, such as heavy metals, have been confirmed to be associated with obesity [3].

Lead (Pb) is a kind of heavy metal widely used in gasoline and lead-acid battery industries [2]. Pb pollutants may be released as air pollution or waste mixtures into soil and waterways in the process of manufacturing, which were then up-taken through food, water and air [4]. Lead poisoning mainly caused brain and neurology defects, namely cognitive disorder, movement and coordination impairment, hearing and visual disturbance [5].Recent studies have suggested Pb as an endocrine disrupting chemical. Pb is not only associated with late-onset puberty [6, 7] but also acts on hypothalamic-pituitary-adrenal (HPA) axis, thus causing high stress-related cortisol levels [8–10]. In addition, high bone turnover is related with Pb exposure [11].Furthermore, a lot of studies have investigated the relationship between early Pb exposure with BMI and obesity, and the results were inconclusive. Some epidemiology as well as animal studies revealed that maternal or adolescent lead exposure caused prolonged obesity that persisted into adults[4, 5, 12, 13], whereas others indicated mother lead level was associated with low birth weight[14, 15]. However, little has concentrated on the effects of adult Pb exposure on obesity. Our previous investigation from the cross-sectional study revealed positive relationship between blood lead level (BLL) and obesity in adults [2]. Thus, it is essential to establish an animal model to explore the underlying mechanisms.

There is a growing recognition of the impact of environmental factors such as heavy metals, diet and stress on the epigenetic regulation of gene expression, especially in early life or at maternal stage [16]. DNA Methylation is one of the most common epigenetic events. Some research has confirmed that both obesity and type 2 diabetes were accompanied by DNA methylation changes of some metabolic-related genes [17–19]. In one study, various levels of maternal lead exposure resulted in the fur color changes of offspring as well as methylation level changes of some gene locus and imprinted genes that determined fur color [4].

In our study, we constructed different levels of leaded water on adult rats, to observe the association of Pb and weight gain and other metabolic parameters. Furthermore, using liver samples, we performed a genome-wide methylome analysis to observe DNA methylation signatures.

## Materials and Methods

### Animal experiments

Twenty-five male 8-week-old Wistar rats were purchased from SLAC Laboratories, SIBS, Shanghai, China. Animals were housed at an ambient temperature of 22 ± 2°C and maintained under a normal 12 hours light/dark cycle and allowed access to food and water ad libitum. After one week of acclimatization, rats were randomly assigned to two groups with one (N = 20) conventionally fed with normal chow diets (NCD; containing 10% fat) and the other (N = 5) with high fat diets (HFD; containing 40% fat), the former group was then divided into 4 sub-groups: NCD (N = 5); NCD+0.05%Pb (N = 5); NCD+0.15%Pb (N = 5); NCD+0.45%Pb (N = 5). Leaded water was made by dissolving Lead (Ⅱ) acetate trihydrate ((CH3COO) 2Pb.3H20) (Sangon biotech, shanghai) in distilled water. Animal treatment lasted for 21 weeks. Body weight and FBG were measured every two weeks at the overnight fasting condition. For the IPGTT, the animals were intra-peritoneally injected with glucose of 2g/kg body weight (Sigma) after 12 h of fasting, and blood glucose levels were measured at baseline and at time points of 15min, 30min, 60min, 90min, 120min after glucose injection by an electronic glucometer (Terumo, Tokyo, Japan). Animals were sacrificed by anesthesia, bones were obtained immediately and bone lead concentration was detected with inductively coupled plasma atomic emission spectroscopy. All the animal experiments were carried out in accordance with the Guidelines for Care and Use of Laboratory Animals of Shanghai Laboratory Animal Center (SLAC), Chinese Academy of Sciences, Shanghai, China. All the protocols were approved by the Institutional Animal Care and Use Committee of SLAC (No. 2011–007). We have made all efforts to minimize animal suffering.

### Measurement of insulin and biochemical indexes

Tail blood was separately collected from the caudal vein following overnight fasting at the end of the experiment for detection of insulin and biochemical profiles. Alanine aminotransferase (ALT), aspartate transaminase (AST), triglycerides (TG), total cholesterol (TC), free fatty acid (FFA) and low-density lipoprotein cholesterol (LDL-C) levels were detected with Siemens Dimension MAX (Siemens Healthcare Diagnostics Inc); FSI was assessed with ELISA kits (Shibayaji, Japan); HOMA-IR index was calculated according to the formula: FBG*FINS/22.5.

### Oil red staining of liver

After the rats were sacrificed, the right lateral lobule of the livers was removed and subsequently fixed in phosphate-buffered 10% formalin. The fixed liver was embedded in paraffin blocks and were sliced and stained with oil red to evaluate lipid droplets.

### Genomic DNA isolation and WGBS library construction

Two samples were randomly selected from control group as well as 0.05%Pb group, separately. Genomic DNA was isolated and purified from 25 mg of frozen liver tissue with the DNeasy Tissue Kit (Qiagen, Germany, cat. no. 51306) according to the manufacturer's protocol. DNA concentration was assessed using a ND1000 spectrophotometer (NanoDrop Technology), and DNA quality was assessed by electrophoresis using a 1% agarose gel. 3ug of genomic DNA were broken into fragments by Covaris S2 system (Covaris, MA) for 52 seconds with 20% duty cycle, level 5 intensity and 200 cycles per burst. Fragmented DNA were purified by Ampure XP Beads (Beckman Coulter, CA) and the fragments were end-repaired, and a single A nucleotide was added to the 3' ends of the fragments in preparation for ligation to a methylated adapter (Illumina, CA) with a single-base T overhang. The ligation products were purified and size-selected (300-400bp) using agarose gel electrophoresis (Qiagen Minelute Gel Extraction Kit). DNA was modified with sodium bisulfite to convert unmethylated cytosine to uracil using the Zymo Gold methylation kit (Zymo Research, CA) according to the manufacturer's protocol and then purified. The purified converted DNA was amplified with PfuTurbo Cx Hotstart DNA Polymerase (Agilent Technologies, CA) and was purified again using AMPure XP beads. Library quality was monitored using the Agilent 2100 Bio-Analyzer (Agilent) and KAPA Library Quantification Kit (Kapa Biosystem). Paired-end sequencing (2×100 bp) was then carried out using the Illumina Hi-Seq 2000.

### Whole-genome methylation analysis

#### Identification of methylated cytosines

After sequencing, raw data were filtered into clean data by removing pollution reads, low quality reads and Adapters. FASTqc software was used to assess sequencing accuracy of the clean data. The sequencing error rates were controlled lower than 5%. Clean data were mapped to the reference genome of rats using in silico bisulfite conversion algorithm with Bismark software. Reads with methylated position were reported when sequencing depth was ≥1. We used the bulk fractional methylation of mitochondria DNA to measure the rate of false positives. The methylation level of an individual cytosine was calculated from the number of sequenced cytosines divided by the total read depth, i.e. (mC)/(mC+non-mC). The methylation level of a region, named “bulk methylation level”, which was calculated from (mC)/(mC+non-mC). mC and non-mC referred to the number of methylated and unmethylated cytosines, respectively, in this region. The cytosine methylation level covered by more than 5 reads were used to calculate DNA methylation levels of whole genome and specific genetic elements such as TTS, TSS, CDS, transposon element etc. TSS was defined as 2000bp of upstream and downstream region of gene start sites. TTS was defined as 2000bp of upstream and downstream region of gene termination site. Methylation signal intensity values of whole genome were drawn in a circular view using CIRCOS visualization software.

#### Identification and enrichment of differentially methylated regions (DMRs) of genes

To identify genes with significant differences in DNA methylation between two groups, we locked in TSS regions of genes. Biological replicates of each group were merged, and afterwards genomic sites with methylation cytosine covered by more than 2 reads were analyzed with DMR caller package of R software. P values were calculated by Fisher’s exact test, and then FDR correction was performed. Cytosines with FDR (<0.05) and methylation differences of at least 0.05 for CG contexts were identified as differentially methylated regions. Single reads of potential DMRs were analyzed using the Integrative Genomic Viewer (IGV) Browser. We processed KEGG pathway enrichment of different methylated genes and calculated the significance of every KEGG pathway with hypergeometric distribution testing method. A small P value indicated differential gene enrichment in specific KEGG pathway.

### Statistics analysis

Data were presented as mean ± SEM. The overall differences between treatment groups were compared using one-way analysis of variance (ANOVA), followed by Tukey’s multiple comparison test, and LSD, with or without repeated measures. P < 0.05 was considered to be statistically significant. GraphPad Prism, Version 5 (GraphPad Software) was used for all statistical analyses.

## Results

### Pb was deposited in femur of rats drinking leaded water for 21 weeks

Table 1 reveals the cumulative bone Pb deposition in each group. Pb was scarcely detectable in femur of both NCD and HFD group. However, significant Pb accumulations were observed in Pb exposure groups (*P* <0.05). The mean Pb content was 56.25±7.47 ng/mg in NCD+0.05%Pb group, which was tripled in 0.15%Pb (160.50±14.15 ng/mg) exposure group. Interestingly, Pb content in NCD+0.45%Pb group (189.30±18.22 ng/mg) did not exhibit the same increasing pattern.

**Table 1:** Bone Pb levels in rats (ng Pb/mg dry wt of femur) at 21 weeks.

	NCD	NCD+0.05%Pb	NCD+0.15%Pb	NCD+0.45%Pb	HFD
Bone lead, ng/mg	≤1	56.25±7.47^a^	160.50±14.15^a^	189.30±18.22^a^	≤1

Data are expressed as mean ± SEM. Pb was determined in thigh bone by inductively coupled plasma atomic emission spectroscopy.

^a^ *P*< 0.05 vs. NCD by one-way ANOVA.

### 0.05%Pb resulted in weight gain

**Fig 1:**
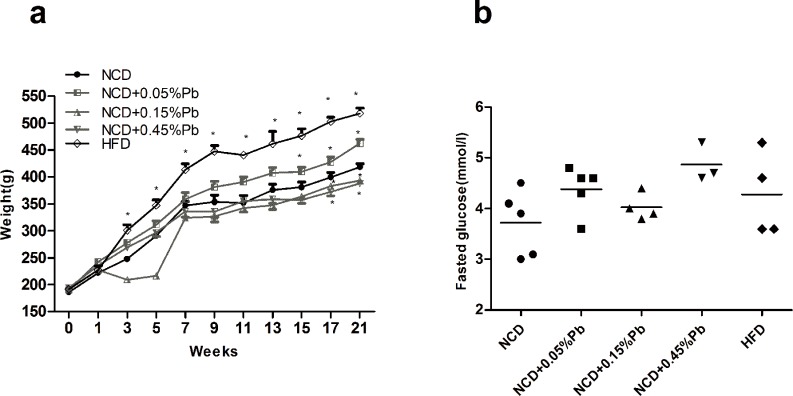
Body Weight and Fasted glucose levels of rats after Pb exposure. (a) Body weight. (b) Fasted glucose level. Data are expressed as mean ± SEM. * *P*<0.05 vs. NCD by one-way ANOVA.

Weight gains (Fig 1A) were observed in all groups. Rats of HFD accumulated significantly more weight than that of NCD at the end of 21 weeks (*P* <0.05). Both the NCD+0.15%Pb and NCD+0.45%Pb groups exhibited less weight gain in comparison with NCD group (P <0.05). Notably, only exposure to 0.05%Pb contributed to increased weight compared with the NCD control group (*P* <0.05).

### 0.05%Pb resulted in glucose intolerance and insulin resistance of rats

**Fig 2:**
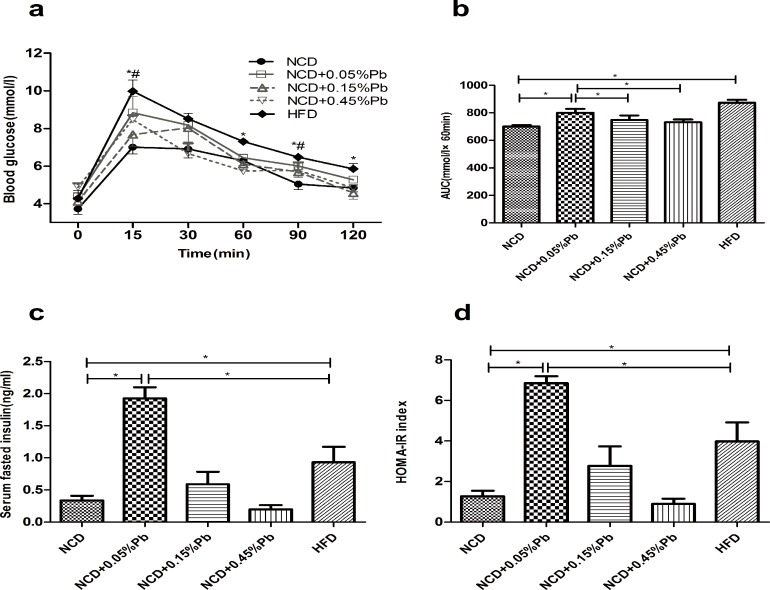
Glucose intolerance and insulin resistance were observed after Pb exposure. (a) IPGTT. * *P*<0.05 HFD vs. NCD; # *P*<0.05 NCD+0.05%Pb vs. NCD by repeated measures data ANOVA. (b) AUC of IPGTT. (c) Fasted serum insulin levels. (d) HOMA-IR indexes. Data are expressed as mean ± SEM. (b), (c), (d)* *P*<0.05 vs. NCD by one-way ANOVA.

Though no significant difference in fasted blood glucose level (Fig 1B) was observed across different groups, in order to evaluate glucose homeostasis, we further conducted IPGTT at the 20^th^ week of the study. The curves of HFD group remained at the top during the 120 minutes (Fig 2A). Meanwhile in the NCD groups, 0.05%Pb resulted in higher peaking glucose level at the 15 min time point compared to controls. To quantify glucose tolerance, we calculated the area under curve (AUC) for all treatment groups. Exposure to Pb increased the average AUC in NCD groups (Fig 2B). Among the NCD groups, the NCD +0.05%Pb group showed a significant higher AUC than NCD control (*P* < 0.05), which was similar to the AUC of HFD. The AUCs of the other two Pb dosages (0.15% and 0.45%) were not significantly larger than that of the NCD control group.

Moreover, a significantly elevated fasted insulin level (FSI) was observed in the NCD+0.05%Pb group (Fig 2C) (P <0.05), which was even higher than in the HFD group (*P* <0.05). The HOMA-IR index exhibited the same patterns (Fig 2D).

### 0.05%Pb led to biochemical changes

**Fig 3:**
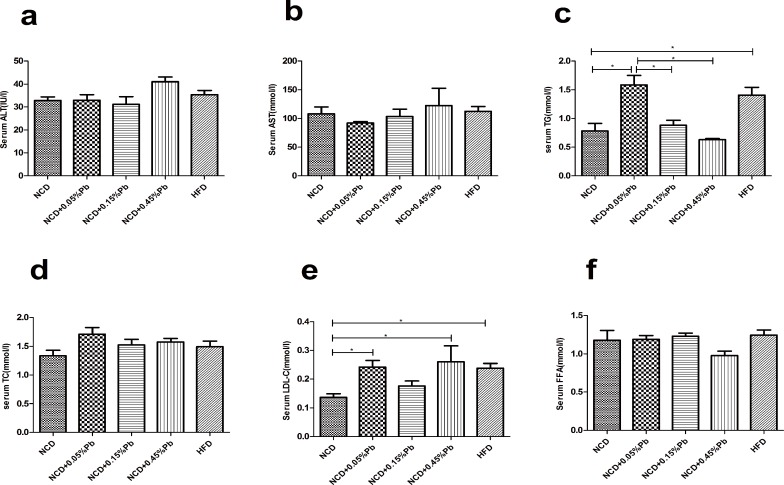
Biochemical changes after Pb exposure. (a)Serum ALT levels; (b) Serum AST levels; (c) Serum TG levels; (d) Serum TC levels; (e) Serum LDL-C levels; (f) Serum FFA levels. * *P*<0.05 by one-way ANOVA.

Alanine aminotransferase (ALT) and aspartate aminotransferase (AST) were detected to evaluate liver function. The NCD+0.45%Pb group showed slightly higher ALT and AST level than others, indicating high Pb dosage exposure might result in liver damage (Fig 3A and 3B), though the differences did not reach significance. The NCD+0.05%Pb group exhibited significantly higher TG level (Fig 3C) (P <0.05) compared to other NCD groups. This group also had higher low-density lipoprotein cholesterol (LDL-C) than NCD control group (*P* <0.05) (Fig 3E). Differences were not observed in serum total cholesterol (TC) and free fatty acid (FFA) levels (Fig 3D and 3F).

### 0.05%Pb led to lipid accumulation of liver

**Fig 4:**
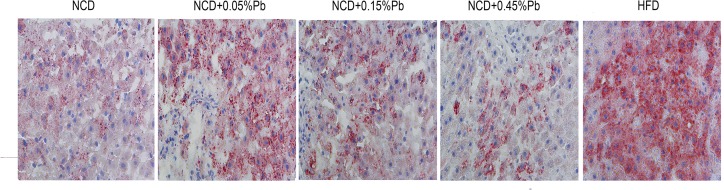
Oil red staining of liver. Lipid accumulation was observed in rats of HFD group as well as exposed to 0.05%Pb. The magnification is 400×.

Oil red staining showed excessive lipid droplets in the hepatocytes of rats fed with high-fat diet (Fig 4). Meanwhile, increased hepatic triglyceride contents were observed in 0.05%Pb treated rats compared to the NCD control rats.

### Whole genome DNA methylation alteration was observed in 0.05%Pb exposed rats

**Fig 5:**
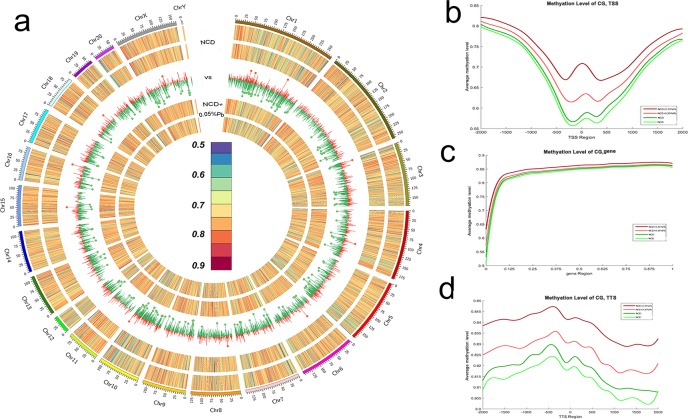
DNA methylation files of liver genome for NCD+0.05%Pb group compared to NCD group. (a) The circus map; (b)The profiling of TSS region; (c) The profiling of gene body region; (d) The profiling of TTS region.

The WGBS data has been uploaded to GEO(Series record:GSE89919).Significant metabolic changes were observed in the NCD+0.05%Pb group, thus we conducted the whole genome bisulfite sequencing of liver samples in both the NCD+0.05%Pb group and the NCD control group. As was observed in the CIRCOS map (Fig 5A), the NCD+0.05%Pb group had hypermethylation at the whole genome level, compared to NCD. The density distribution curves revealed that unmethylated CG sites were reduced and 100% mCG sites were increased after 0.05%Pb treatment (S1 Fig). Furthermore, average DNA methylation levels were all elevated in specific genetic functional elements such as gene, intergenic, intron, extron, CpG Island as well as repeated elements (S2 Fig). The profiling analysis revealed the overall trend of DNA methylation in different regions of gene, transcription start site (TSS) and transcription terminal site (TTS) sections (Fig 5B–5D). All the three sections showed increased methylation levels at overall length, although there were small differences between two samples of the same group.

### Differentially methylated genes associated with glucose and lipid metabolism in 0.05%Pb exposed rats

**Fig 6:**
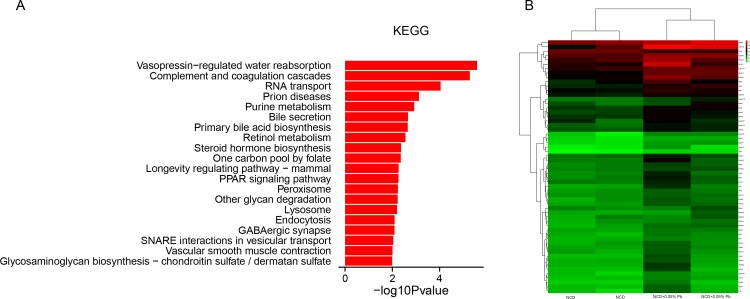
DNA methylation enrichment and specific genes with altered DNA methylation for NCD+0.05%Pb group compared to NCD group. (a) The top 10 KEGG pathways changed in NCD+0.05%Pb group compared to NCD group. *P* values were derived from Fisher’s Exact Test. (b) The heat map of specific genes related with glucose and lipid metabolism (FDR<0.25).

KEGG pathway enrichment analysis was employed to identify important pathways that were altered in Pb exposure group compared to control group (Fig 6A). Among the top 20 pathways with altered DNA methylation were metabolic pathways, fatty acid elongation pathway as well as TCA cycle pathway.

Difference analysis in gene methylation aimed at TSS region indicated that a total of 2733 genes experienced DNA methylation changes, with 2320 hypermethylated genes and 413 hypomethylated genes (S1 Table). A heatmap of different methylated genes related to lipid and glucose metabolism were employed (Fig 6B). DNA hypermethylation was observed in genes related to fatty acid transport and synthesis (LDLR, FATP, Glyctk, DGAT1 and DGAT2) and fatty acid oxidation (CPT-2, MCAD, CYP4A, ACO). In addition, genes related to VLDL-TG assembling (MTTP and APO-CⅢ, APO-, APO-Ⅳ) and the transcription factor such as LXR and SREBP-1c were hypermethylated. Some glucose metabolism related genes such as PKA and AKT were hypermethylated. PEPCK (1.45 multiple) and G6pase (1.18 multiple) related with gluconeogenesis were also hypermethylated, as well as cholesterol metabolism genes such as CYP51 and ACAT.

## Discussion

In our research, we found that 0.05%Pb water exposure brought about weight gain and insulin resistance as well as glucose intolerance in the NCD rats, accompanied by elevated serum TG as well as hepatic lipid accumulation, which were not seen in the other two dosages (0.15% and 0.45%) of NCD groups. Furthermore, compared to the NCD control group, we found the DNA hypermethylation extended along pathways related to glucose and lipid metabolism in the NCD+0.05%Pb group. These results indicated that 0.05%Pb may induce abnormal metabolic status through DNA methylation that regulated important genes involved in lipid and glucose metabolism.

As a kind of environmental endocrine disruptor, Pb mimics or disrupts hormone function at low doses in ways not predicted by high-dose studies [20]. Previous animal studies had found that Pb exposure played both dose-specific and sex-specific roles in body weight and glucose dys-metabolism, however, most of their studies focused on prenatal Pb exposure, and the effective dose and conclusions were discrepant. In one study, prenatal exposure to 16ppm and 32ppm lead water caused weight gain in male offspring of mice; moreover, body fat and FSI level as well as HOMA-IR level of 16ppm male offspring were significantly increased. In contrast, weight gain and body composition changes as well as glucose intolerance were not seen in female offspring [12]. In addition, a linear increase in mean weight with increasing pre-weaning lead exposure in male offspring was observed in a study constructed by Faulk et al. [4].

The effects of postnatal Pb exposure on metabolism changes were inconclusive. Our previous epidemiology study revealed a positive relationship between BLL and BMI in China [2]. In another longitudinal study in Boston, the chronic lead exposure in childhood may result in obesity that persists into adulthood [13]. Whereas Both Miguel A. Padilla et al. and Franco Scinicariello et al. reported that BLL associated with lower body weight in adults based on data from NHANES [1, 21]. In another study, no association was found between BLL and BMI in adults [22]. The conflicting results may be due to ethnic variations as well as different BLL of the study population. In one animal study, gestational exposure from 27 to 109ppm of leaded water resulted in late-onset obesity of offspring, which was not observed in the same-dosage treated postnatal groups[5]. In another animal study constructed by Eric Beier et al. [11], postnatal 50ppm leaded water ingestion had no effect in either body weight or fat composition of mice, whereas it caused higher fasted glucose level and elevated leptin levels.

Gestational exposure has more detrimental effects on offspring, probably due to high efficiency of placenta absorption; thus postnatal Pb exposure in rats may achieve equivalent effect as in prenatal ones only when increasing the exposure dosage. Therefore, higher concentration basis and gradients were set in our experiment compared to previous studies [5, 11].

In our experiment, weight gain was observed in the NCD rats received 0.05% lead acetate, which was partly in accordance with our previous epidemiology study [2] Glucose tolerance was also impaired when exposed to Pb, which can be resulted from defects in insulin secretion and/or insulin sensitivity. Both fasting insulin and glucose levels were elevated in the NCD+0.05%Pb group, indicating excessive hepatic output of glucose and/or insensitivity of peripheral tissues especially liver, which could also be verified by HOMA-IR index. Therefore, it can be speculated that lead may disrupt glucose homeostasis, somewhat, through induction of insulin resistance. Mostafalou et al. [23]also found that 0.05% Pb level of drinking water could lead to glucose intolerance and insulin resistance in rats. Whereas, overweight itself was a contributing factor to hepatic lipid content and insulin resistance as well as glucose intolerance, all of which could be observed in 0.05% Pb received groups, thus it was hard to illustrate whether lead acted mainly as an “obesogen” or directly devoted to insulin resistance.

DNA methylation is an important style of epigenetic regulation [24]. Previous studies have concentrated on the relationship between Pb exposure and the methylation changes of brain. Developmental Pb exposure not only altered DNA methylation in both specific gene level and global genome level in brain of Alzheimer’s disease model [25–28], but also could affect the expression of DNA methyltransferase 1(DNMT1) and DNA methyltransferase 3a (DNMT3a) in hippocampus [29]. In addition, Pb exposure could enhance the mean DNA methylation level and epigenetic drift of several imprinted genes like IGF-2, Igf2r, H19 in both cell model and animal tissues [30, 31], although others revealed that increased blood or patella lead levels were associated with hypomethylation of LINE-1 in both cell model and population study[32, 33]. Furthermore, Arko *et al*. found that Pb exposure during pregnancy affects the DNA methylation status of the fetal germ cells, which led to altered DNA methylation in grandchildren’s neonatal dried blood spots [34].

The liver is a critical organ for the regulation of whole body energy homeostasis due to its central role in lipid and glucose metabolism, as well as its close connection via the portal vein to nutrient uptake in the intestine [17]. Epigenetic mechanisms may partly explain the pathogenesis and development of metabolic diseases, as previous studies has found altered DNA methylation accompanied by altered transcriptional levels of liver in obesity and type 2 diabetes. Therefore, we performed a global wide analysis of DNA methylation in liver to explain the mechanism of metabolic changes resulting from adult Pb exposure for the first time. The bisulfite sequencing results revealed a hepatic hypermethylation of whole genome of 0.05% Pb exposed rats compared to unexposed ones, which may be explained by changed expression or activity of proteins controlling DNA methylation [18]. In addition, a lot of genes involved with glucose and lipid metabolism were observed with apparent DNA methylation alterations at the TSS regions [35]. PKB/AKT pathway was essential in regulating lipid and glucose metabolism in liver. Hypermethylation of AKT may result in gene repression and thus inhibiting this pathway, which could lead to reduced glucose uptake and glycogen synthesis but increased gluconeogenesis, thus promoting insulin resistance of liver [17]. The hypermethylation of genes involved in fatty acid oxidation such as CPT-2, MCAD, and ACO could result in reduced fatty acid oxidation rate and in turn elevated lipid synthesis in liver. MTTP and lipoprotein like APO-CⅢ, APO-Ⅴ, and APO-Ⅳ were important in VLDL-assembling and TG transportation. Hypermethylation of these proteins may give rise to reduced TG output and a further accumulating of TG in liver [36].

Bone lead level is a more reliable biomarker of long-term Pb exposure, while blood lead level reveals recent exposure [15]. Thus, we evaluated Pb accumulation by detecting bone lead level instead of blood lead level, which was differed from most previous studies [12, 21]. Whereas, it would be more rigorous to detect both BLL and bone lead level. Furthermore, the lead level of NCD+0.45% group did not cause corresponding increase in bone lead levels, which may due to the precipitation of the highest concentration of lead in the water.

Our study has some merits. Firstly, for the first time, we performed whole genome DNA methylation analysis of liver in both Pb exposure rats and control rats, in order to explore the potential mechanism of metabolic changes after exposure to lead. Secondly, our study concentrated on adult stage but not prenatal or developmental stage. This made good sense in our daily life, because the metabolic disorder and altered DNA methylation of adults may have adverse effects on the next generation.

In conclusion, we found that chronic 0.05%Pb exposure results in dose-specific insulin resistance and weight gain. These metabolic disorders may be induced through altered methylation of genes related with metabolism. Further studies are warranted to explore the alteration of specific gene expression and its association with DNA methylation in both animal and cellular levels.

## Supporting Information

S1 Fig The density distribution curves of methylated CG sites. (a)(b) NCD+0.05%; (c)(d) NCD.
(TIF)

S2 Fig Average DNA methylation levels in specific Genetic functional elements. (a)(b) NCD+0.05%; (c)(d) NCD.
(TIF)

S1 Table Differential methylated genes. proportion1: NCD; proportion2: NCD+0.05%Pb.
(XLS)
