# Supplementary figures and images for: Lead Exposure Induces Weight Gain in Adult Rats, Accompanied by DNA Hypermethylation
Source: PLoS One. 2017 Jan 20;12(1):e0169958. doi: 10.1371/journal.pone.0169958 (PMC5249225; doi:10.1371/journal.pone.0169958)

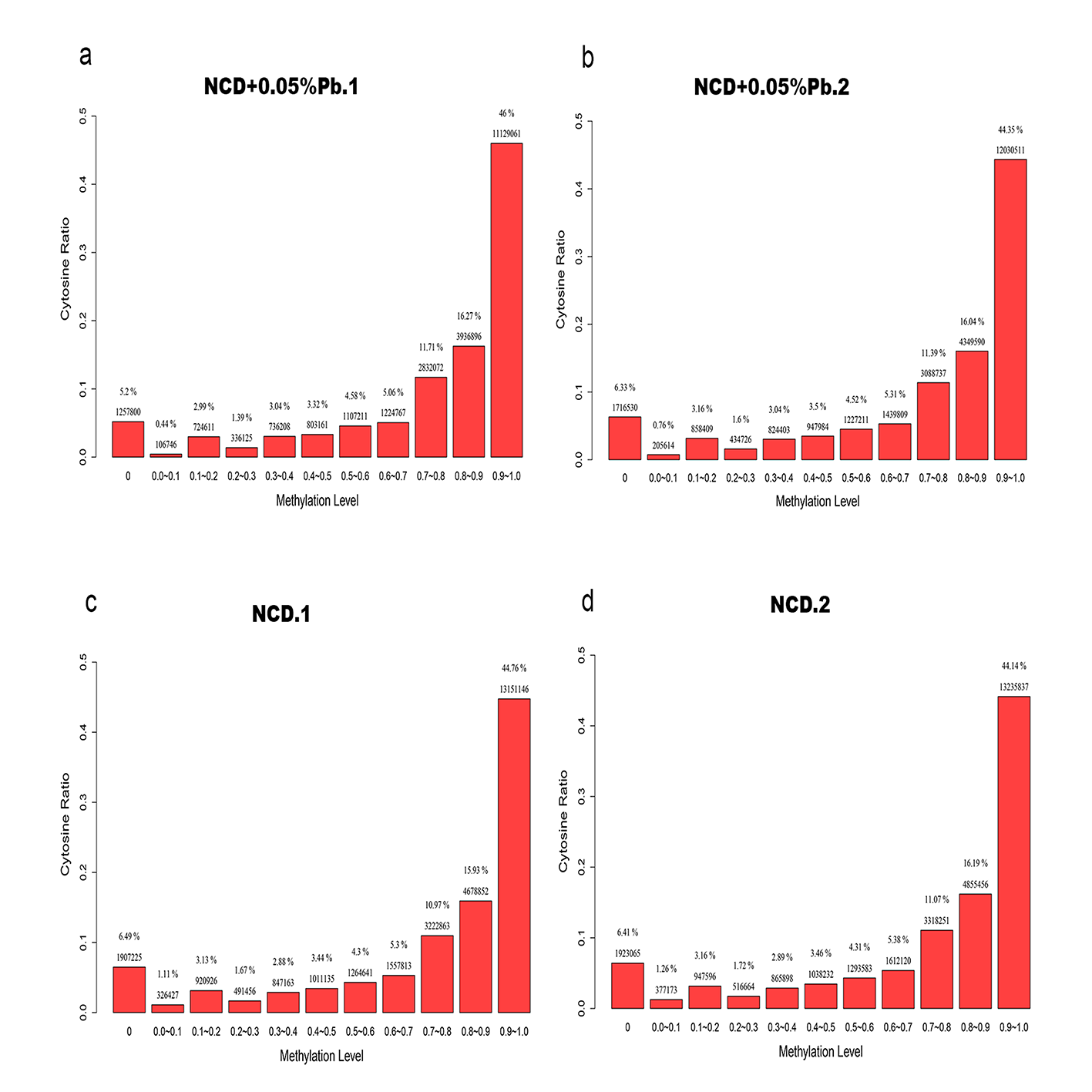

Supplement: S1 Fig — (a)(b) NCD+0.05%; (c)(d) NCD. (TIF) [file pone.0169958.s001.tif]

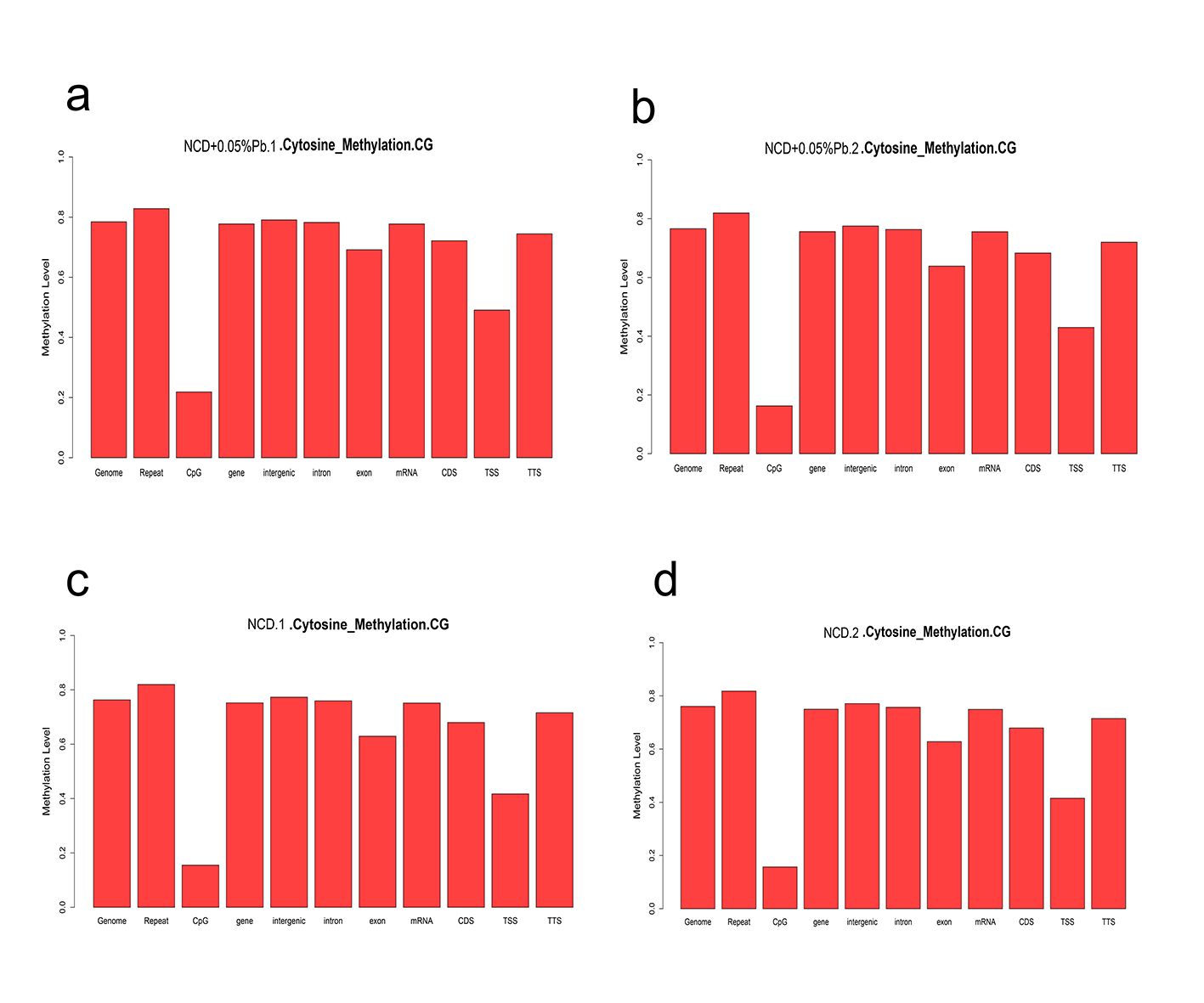

Supplement: S2 Fig — (a)(b) NCD+0.05%; (c)(d) NCD. (TIF) [file pone.0169958.s002.tif]
